# Photographic Evaluation of Burn Depth via Telemedicine: Insights from Iranian Surgeons

**DOI:** 10.1089/tmr.2023.0048

**Published:** 2023-09-13

**Authors:** Hamed Shahdadi, Somayeh Rezayi, Fatemeh Shahrahmani, Ali Akbar Mohamadi

**Affiliations:** ^1^ Department of Surgery, Faculty of Medicine, Shiraz University of Medical Science, Shiraz, Iran.; ^2^ Department of Nursing, Faculty of Nursing and Midwifery, Islamic Azad University of Khorasgan, Isfahan, Iran.; ^3^ Department of Surgery, Faculty of Medicine, Mashhad University of Medical Science, Mashhad, Iran.; ^4^ Department of Surgery, School of Medicine, Shiraz University of Medical Science, Shiraz, Iran.

**Keywords:** burn, surgeons, photograph, telemedicine

## Abstract

**Background:**

The accurate assessment of burn depth is crucial for determining appropriate treatment. Telemedicine has emerged as a promising tool for supporting burn diagnosis and decision-making, as it allows for remote consultation with burn specialists and access to high-quality imaging. The aim of this study was to evaluate the diagnostic capabilities of telemedicine in diagnosing burn depth.

**Methods:**

A total of 15 Iranian surgeons participated in this study; they were presented with 13 images of partial thickness burn ulcers located on the extremities and trunk of patients. The participating surgeons were required to provide their diagnoses of burn type and depth, as well as the necessity of surgical intervention, and their responses were recorded.

**Results:**

Data from 11 participants and 143 responses were analyzed. The average diagnostic accuracy for superficial burns was 79.3%, while for deep burns, it was 13.72%. The mean total diagnostic accuracy was 75.2%.

**Conclusion:**

The results of this study suggest that photographs can be a reliable diagnostic tool for evaluating superficial burns. However, photographs are neither valid nor reliable for assessing burn depth. These findings have important implications for the use of telemedicine in burn diagnosis and indicate that additional diagnostic tools may be necessary for accurate assessment of deep burns.

## Introduction

Burn injuries are a significant global health concern, affecting millions of people every year, causing long-term disabilities and mortality, particularly in developing countries where access to health care and resources may be limited.^1^ The physical and psychological effects of burns are significant, and their treatment imposes high costs on the health care system and society.^2,3^ Effective diagnosis and treatment of burns are crucial to prevent long-term disabilities, reduce mortality, and minimize the economic burden on individuals and society.^4–6^

Two treatment strategies are employed, namely partial injuries, which include superficial thickness (epidermal), superficial-partial thickness (superficial dermal) burns that will need conservative treatment, and deep injuries, which include deep-partial thickness (deep dermal) and full thickness (sub dermal) burns that require surgical intervention and skin grafting to heal.^7–9^

However, distinguishing between superficial-partial and deep partial-thickness burns is particularly challenging and crucial because the latter requires more complex and specific treatments.^5,10,11^

In recent years, telemedicine has emerged as a promising tool for remote diagnosis and consultation, particularly for skin conditions such as burns.^12–14^

In burn care, telemedicine has been used to improving diagnostic accuracy, reducing unnecessary referrals, facilitate remote consultations, and enabling earlier intervention and treatment.^10,15^ Furthermore, telemedicine can provide a cost-effective alternative to in-person consultations, especially for patients who live in remote or underserved areas.^16,17^

Photography is one telemedicine tool that has been used to aid in burn diagnosis. The use of photographs can provide an accurate, objective record of the burn wound that can be shared remotely with health care providers for diagnosis and management planning.^12,18,19^ Previous studies have suggested that photographic assessment can be a reliable tool for evaluating superficial burns. However, the reliability of photographic assessment for determining burn depth is less clear.^12,18,20,21^

The aim of this study is to evaluate the diagnostic capabilities of telemedicine, including photographic assessment, in accurately determining the depth of burns, with a particular focus on partial thickness burns which pose a significant challenge even for experienced clinicians. In deep burns, early intervention can reduce the progression of scarring and its complications, while incorrect assessments of burn depth can lead to unnecessary surgery. The study was conducted on a sample of Iranian patients, with the goal of improving burn diagnosis and treatment options, as well as facilitating appropriate referrals for grafts where necessary.

## Methods

This cross-sectional study aimed to evaluate the diagnostic accuracy of telemedicine in assessing burn depth, specifically through photographic evaluation by Iranian surgeons. The study included a total of 15 surgeons actively working in the field of burns. The selection of participants was conducted using an available sampling method, with inclusion criteria requiring surgeons to have at least 2 years of active experience in the field of burns. Before the commencement of the study, informed consent was obtained from all 13 patients, granting explicit permission for the documentation of photographs. The consent process was in compliance with the guidelines and regulations set forth by the appropriate human subjects committee, ensuring the ethical conduct of the research. The study was approved by a human subjects committee in Shiraz University of Medical Sciences.

The sample size was determined using the sample-size estimation formula, which calculated the required number of participants to estimate the success ratio with a confidence level of 90% and a confidence interval of 0.08, based on an accuracy rate of 80% observed in similar studies.

**FIG. 1.**
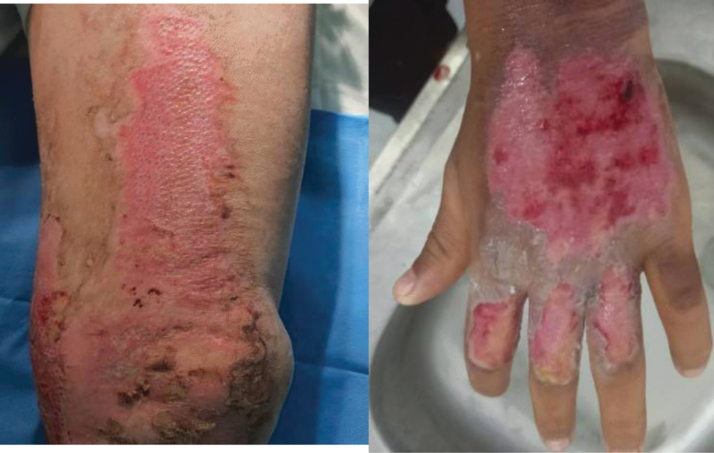
examples of cases presented in the study.

Thirteen standardized images of partial thickness burns located on the extremities and trunk of burn patients were presented to the participating surgeons (Fig. 1). There were six deep images vs. seven superficial. Out of the 15 participants involved in our study, 11 surgeons answered all questions, and data analysis was conducted using their responses.

All images were taken within 24 h of the burn and before any interventions, captured using a standardized digital camera (Canon EOS 1300D 18–15 mm DC III Digital Camera) at Shiraz Burn Accident Center in 2017–2018. We endeavored to adhere to the American Telemedicine Association guidelines for image acquisition. This involved utilizing a high-quality camera to capture medical images, ensuring the protection of patient privacy during the image acquisition process. Before capturing any medical images, patients were required to provide informed consent explicitly permitting the use of their images for diagnostic or clinical purposes. Additionally, we applied standardized image capture techniques to minimize distortions or artifacts that could potentially impact the diagnostic accuracy of the images.

The photographs were displayed on a full high definition (HD) computer screen for each surgeon to diagnose the type and depth of the burn and asked if the burns required a graft or not, and the resulting diagnoses were recorded. The resolution of the display was 1920 × 1080 pixels (Full HD or 1080p). Microsoft Photos software was used to display the images. Users can zoom and pan the image to view different areas of interest. However, they cannot adjust the color scale. They had no information on patient disease, type of burn, final treatment, and patient outcome.

The accuracy of the diagnoses made by participating surgeons was compared with the diagnoses recorded in the patients' medical records, which were confirmed by initial diagnosis of an independent expert through clinical examination at the time of admission and final treatment.

Data analysis was conducted using SPSS version 20 software, utilizing independent *t*-tests and chi-square analysis to assess statistical significance.

## Results

Out of the 15 participants involved in our study, 11 surgeons answered all questions, and data analysis was conducted using their responses. The average working experience was 7.81, and the maximum work experience was 18 years, and the minimum was 3 years. In total, 143 responses were analyzed, as each participant evaluated 13 photos. The average diagnostic accuracy for superficial burns was 79.3%, while for deep burns that needed surgical interventions, it was 13.72%. The mean total diagnostic accuracy across all burn depths was 75.2%. These findings suggest that telemedicine can be a useful tool in assessing superficial burns, but may be less reliable in identifying deep burns.

A General Linear Model and Repeated Measures analysis of variance were utilized to investigate the impact of work experience on diagnostic accuracy. Based on the findings, work experience did not have a significant effect on the estimation of burn accuracy, including both overall accuracy (*p* = 0.189) and accuracy for superficial burns (*p* = 0.192) and deep burns (*p* = 0.098) (Table 1).

**Table 1.** Work Experience

	Deep/superficial	Location (extremity/trunk)	Correct answer
Pic 1	D	E	1
Pic 2	S	T	9
Pic 3	D	T	1
Pic 4	D	T	2
Pic 5	S	E	10
Pic 6	S	E	8
Pic 7	D	E	1
Pic 8	D	T	2
Pic 9	S	T	8
Pic 10	S	T	11
Pic 11	S	E	8
Pic 12	S	E	7
Pic 13	D	E	2

**FIG. 2.**
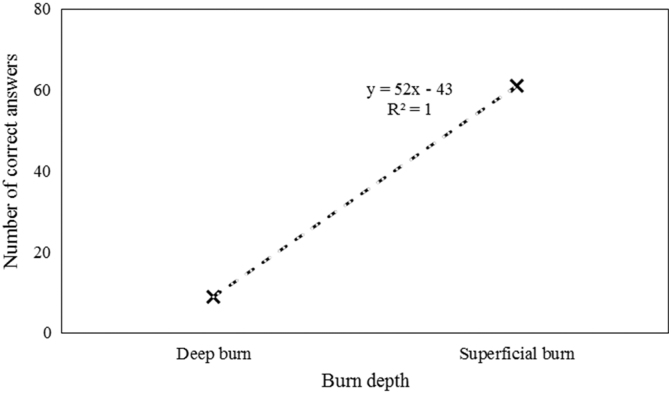
The relation of burn depth and number of right answers.

The following figure shows the relation of burn depth and number of right answers (Table 2; Fig. 2).

**Table 2.** Pictures Location and Correct Answers

Mean	7.81
Less than 5 years	4
Between 6 and 10 years	6
More than 10 years	1

## Discussion

The results of our study indicate that telemedicine, specifically photographic evaluation, is a reliable tool for diagnosing superficial burns, while its accuracy is lower for detecting deep burns, clinical evaluation is the primary method for determining the depth of burn injuries, which are critical for guiding treatment decisions.^5,9^ However, it's important to note that the visual aspect of the burn is the primary factor in determining its depth, and a photograph cannot account for other clinical elements such as sensitivity, tissue retraction, hair adhesion, bleeding, or scarification that may impact clinical diagnosis.^9,22^

Previous research has indicated that the accuracy of burn diagnosis based on photographs can vary depending on the characteristics of the patients and the backgrounds of the clinicians involved. For example, Boissin et al.^23^ conducted a study focused on patients with darker skin types and reported an average accuracy of 67.5% and 66% in diagnosing burn size and depth, respectively. This study found higher accuracy in detecting burn depth.

On the other hand, a study by Blom et al.^18^ involving burns specialists from South Africa and Sweden, as well as emergency medicine specialists from South Africa, found high accuracy in assessing total body surface area using image-based methods, but low accuracy in diagnosing burn depth. However, these results suggest that while photographs can be useful in diagnosing burn size, they should not be the sole method for assessing burn depth.

In another study, Boccara et al.^5^ reported that photographic evaluation of burn depth was comparable to clinical evaluation in 76% of cases overall. In this study, the rate of errors was low in the most superficial and the deepest burns (11% and 13%, respectively), but high in intermediary burns (29.6%). In our study, we focused exclusively on partial thickness burns. This decision was made because, as Boccara's study showed, the diagnosis of depth for these types of burns is known to be very controversial and challenging.

Additionally, Hop et al.^12^ conducted a study examining the reliability and validity of utilizing burn photographs to evaluate burn size and depth. Their findings indicated that burn size can be reliably and validly assessed using photographs of the burn wound, whereas burn depth cannot. In our study, we also observed that the accuracy of photographs in detecting the depth of burns was not reliable. Furthermore, the use of photographs to distinguish between conservative and surgical treatment options could not be deemed valid. This may be attributed to the absence of other clinical elements, such as tissue sensitivity, retraction, and scarification. It is possible that these factors play an essential role in determining appropriate treatment options, which cannot be adequately captured through photographs alone.

Overall, the results of our study and previous research suggest that telemedicine, including photographic evaluation, can be a useful tool in diagnosing superficial burns. However, its accuracy may be limited when it comes to diagnosing deep burns. Further research is needed to explore the potential limitations and benefits of telemedicine in burn assessment and to develop more accurate and reliable methods of diagnosing burn depth.

## Conclusion

This study evaluated the diagnostic capabilities of telemedicine in burn diagnosis, specifically the accuracy of photographs in assessing burn depth. The findings indicated that while photographs can be a reliable tool for diagnosing superficial burns, their accuracy is limited in assessing deep burns. The study highlights the importance of using additional diagnostic tools in burn assessment and decision-making, especially in cases of deep burns where accurate assessment is crucial for determining appropriate surgical treatment. These results have important implications for the use of telemedicine in burn diagnosis and suggest the need for further research to explore the potential limitations and benefits of telemedicine in burn assessment, as well as to develop more accurate and reliable methods of diagnosing burn depth. Ultimately, the goal is to improve patient outcomes and provide optimal care for burn patients.

## Declaration

Authors confirm that their research is supported by an institution that is primarily involved in education or research.
